# Tracking of Glycans Structure and Metallomics Profiles in *BRAF* Mutated Melanoma Cells Treated with Vemurafenib

**DOI:** 10.3390/ijms22010439

**Published:** 2021-01-04

**Authors:** Monika K. Nisiewicz, Agata Kowalczyk, Anna Sobiepanek, Agata Jagielska, Barbara Wagner, Julita Nowakowska, Marianna Gniadek, Ireneusz P. Grudzinski, Tomasz Kobiela, Anna M. Nowicka

**Affiliations:** 1 Faculty of Chemistry, University of Warsaw, Pasteura Str. 1, PL-02-093 Warsaw, Poland; mnisiewicz@chem.uw.edu.pl (M.K.N.); akowalczyk@chem.uw.edu.pl (A.K.); ajagielska@chem.uw.edu.pl (A.J.); barbog@chem.uw.edu.pl (B.W.); mgniadek@chem.uw.edu.pl (M.G.); 2 Faculty of Chemistry, Warsaw University of Technology, Noakowskiego Str. 3, PL-00-664 Warsaw, Poland; asobiepanek@ch.pw.edu.pl; 3 Biological and Chemical Research Centre, Faculty of Chemistry, University of Warsaw, Zwirki i Wigury Str. 101, PL-02-093 Warsaw, Poland; 4 Laboratory of Electron and Confocal Microscopy, Faculty of Biology, University of Warsaw, Miecznikowa Str.1, PL-02-096 Warsaw, Poland; julita@biol.uw.edu.pl; 5 Faculty of Pharmacy, Medical University of Warsaw, Banacha Str. 1, PL-02-097 Warsaw, Poland

**Keywords:** glycans structure, metallomics profile, melanoma cells with *BRAF* mutation, vemurafenib, *α*1-acid glycoprotein

## Abstract

Nearly half of patients with advanced and metastatic melanomas harbor a *BRAF* mutation. Vemurafenib (VEM), a BRAF inhibitor, is used to treat such patients, however, responses to VEM are very short-lived due to intrinsic, adaptive and/or acquired resistance. In this context, we present the action of the B-Raf serine-threonine protein kinase inhibitor (vemurafenib) on the glycans structure and metallomics profiles in melanoma cells without (MeWo) and with (G-361) *BRAF* mutations. The studies were performed using *α*1-acid glycoprotein (AGP), a well-known acute-phase protein, and concanavalin A (Con A), which served as the model receptor. The detection of changes in the structure of glycans can be successfully carried out based on the frequency shifts and the charge transfer resistance after interaction of AGP with Con A in different VEM treatments using QCM-D and EIS measurements. These changes were also proved based on the cell ultrastructure examined by TEM and SEM. The LA-ICP-MS studies provided details on the metallomics profile in melanoma cells treated with and without VEM. The studies evidence that vemurafenib modifies the glycans structures and metallomics profile in melanoma cells harboring *BRAF* mutation that can be further implied in the resistance phenomenon. Therefore, our data opens a new avenue for further studies in the short-term addressing novel targets that hopefully can be used to improve the therapeutic regiment in advanced melanoma patients. The innovating potential of this study is fully credible and has a real impact on the global patient society suffering from advanced and metastatic melanomas.

## 1. Introduction

Glycosylation plays a key role in protein folding, stability, and function. The changes in the structure of glycans are characteristic of many pathologies, including cancer, bacterial adhesion, viral infection, or inflammation [1,2,3]. Glycans expressed at cell plasma membrane surface of all cell types act as recognition elements in cell–cell and cell–extracellular matrix interactions [4]. Cell membrane surface glycans are linear or branched oligosaccharides covalently attached to the cell membrane proteins or lipids. They are biosynthesized and processed by a series of enzymes in a non–template-directed manner, giving rise to heterogeneous glycoforms that impact ligand–receptor interactions [5,6]. Since they accompany the formation of metastases, they are often applied as cancer progression markers [7]. The crucial role of specific, modified glycans in various phases of melanoma progression has documented that changes comprise the increase in the amount of hypersialylated *N*-oligosaccharides or the presence of short, simple glycans. These properties determine their interactions with specific lectins, which is used for the identification and estimation of created complexes. Glycoproteins are also present in biological fluids as soluble molecules released by specific cells performing various functions under physiological and pathological conditions. One such important glycoprotein is the *α*1-acid glycoprotein (AGP), also known as orosomucoid. It belongs to the family of acute-phase proteins produced mainly in the liver in response to inflammation and has an anti-inflammatory and immunomodulatory role in all mammals [8]. Alterations in the human plasma protein levels of AGP have been well documented for numerous physiological and pathophysiological conditions including lung and breast cancer and malignant mesothelioma [9]. The AGP structure consists of about 45% of five *N*-linked glycans, which differ in the degree of branching [10,11]. It has been proven that cancer patients showed, on average, a significantly higher serum AGP level compared with healthy controls [12]; however, this higher level is not correlated with cancer type but rather with its stage [13]. It was also shown that in the cancer patients, not only the total concentration of AGP, but its glycosylation pattern is also altered [2,14]. The direct relationship between immunomodulatory properties of AGP and the degree of glycans branching has been proven, for now, only in the case of breast cancer [15].

One way to detect changes in the glycan structure is to monitor the interaction of specific lectins with glycans. Lectins are the family of highly diverse proteins of animal, plant, and fungus origin, containing domains that selectively recognize and reversibly bind with extremely high binding affinities to the respective types of glycans without altering their structure. Moreover, they may be applied as tools for the distinction between malignant and non-malignant cells. The majority of lectins applied for the recognition and identification of the primary from metastatic tumor cell lines are of plant origin [16]. Lectins isolated from *Canavalia ensiformis* (Con A), *Sambucus nigra* (SNA-1), *Lens culinaris* (LcL), *Maackiaamurensis* (MAA), and animal agglutinin *Helix pomatia* (HPA) are the most frequently used. It has been documented that among them, Con A binds specifically glycans of cancer cells and does not react with normal cells. Note that Con A belongs to the mannose-specific lectins that recognize mannose-containing glycans, single mannose molecules, and the larger mannose-containing *N*-glycan chains that commonly are expressed at the surface of various cancer cells [17,18,19,20]. The study of the interaction between different melanoma cell lines and Con A lectin performed in a concentration-dependent manner with QCM-D showed statistically significant differences in the affinity dependent on the cell phenotypes due to stages of melanoma progression [21].

Numerous published evidence shows that protein glycosylation can be affected by environmental factors, e.g., temperature, pH, dissolved oxygen, or even some chemicals [22,23,24,25]. Here, we report, to our knowledge for the first time, that vemurafenib (VEM), an inhibitor of the B-Raf serine/threonine protein kinase, is enabled to modify the glycans structure influencing the metallomics profile in *BRAF* mutated melanoma cells. Treatments with Con A used as the model receptor, allowed to elucidate the influence of VEM on the glycan structures. The studies were performed on secreted α1-acid glycoprotein (AGP) using quartz crystal microbalance with dissipation (QCM-D) and electrochemical impedance spectroscopy (EIS). The application of these two techniques, which are based on two different working principles, allowed exploring and deeply looking into the AGP-VEM interactions from a broader perspective. Because the degree of *N*-glycan branching and extent of terminal fucosylation can attenuate or enhance protein-drug interactions [26], the important part of our studies was the investigations of melanoma cell lines with (G361) and without (MeWo) *BRAF* mutation treated with VEM. We aim to focus on different metallomics profiles in these cells, trying to find a new role of the surface membrane glycans and transmembrane-intracellular signals addressing the resistance phenomenon frequently observed in malignant melanoma harboring multiple *BRAF* mutation. To data, the comprehensive measurements of cell characteristics were carried out using scanning (SEM) and transmission (TEM) electron microscopy, inductively coupled plasma mass spectrometer with laser ablation (LA-ICP-MS), and quartz crystal microbalance with dissipation energy monitoring.

## 2. Results and Discussion

### 2.1. Influence of Vemurafenib on Glycoprotein Structure

**Figure 1:**
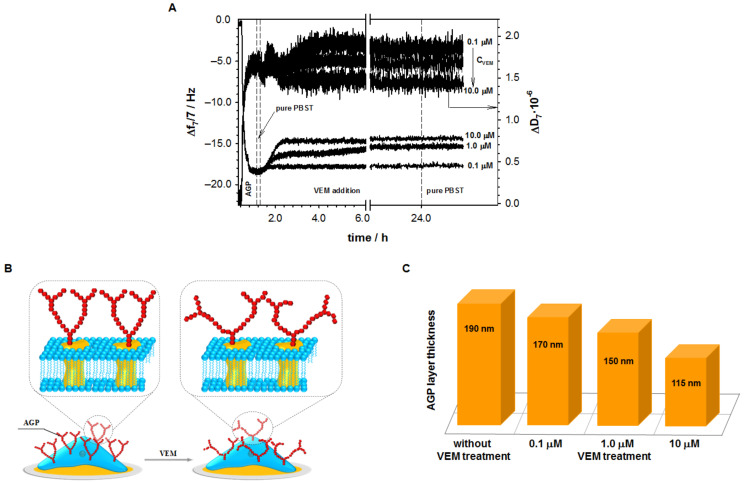
(**A**) Frequency shift observed with AGP and VEM injection to the PBST solution with the addition of 1% DMSO; (**B**) scheme of VEM action versus AGP; (**C**) changes in AGP layer thickness caused by VEM.

To get the precise information about the influence of vemurafenib on the glycoprotein structure, the experiments were performed with glycoprotein (AGP) and concanavalin A (Con A) as receptor models. The changes of the AGP structure were recorded on the basis of the efficiency of its interaction with lectin Con A. The measurements were carried out in the AGP layer treated with and without VEM (24 h) using both quartz crystal microbalance with dissipation and electrochemical impedance spectroscopy, respectively. Figure 1A shows the influence of VEM on the AGP layer. The experiments were performed with three different VEM concentrations. The increase in the frequency shifts proved that the structure of AGP changed (Figure 1B) after its interaction with VEM. Moreover, this effect was strongly dependent on VEM concentration; the higher the VEM concentration, the higher the increase of Δ*f*. It is obvious that the change in the AGP structure forces the reorganization of the glycoprotein layer and thus changes its properties such as viscoelasticity or hydration. Moreover, the change in the glycoprotein structure must affect its layer thickness. The AGP layer thickness decreased with increasing VEM concentration (Figure 1C). For the highest applied VEM concentration (10 μM), the AGP layer thickness decreased ca. 40% compared to the initial value (AGP layer untreated with VEM). Most likely, the reduction in the thickness of the layer is the result of its denser packing resulting from the rearrangement of the glycan chains. The increase in the packing density is also very visible in the values of charge transfer resistance (*R*_ct_) and double layer capacity (*C*_dl_) estimated from EIS measurements. The exposure of AGP layer to VEM solution led to an increase in the *R*_ct_ and a decrease in *C*_dl_ value, as shown in Table 1.

**Figure 2:**
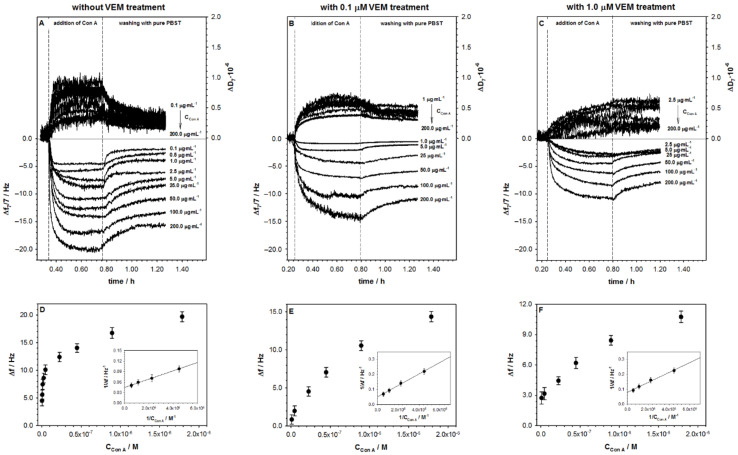
(**A**) Typical QCM-D spectra of the shifts in frequency (Δ*f*) and dissipation factor (Δ*D*) recorded during covalent binding of Con A to AGP without, and (**B**,**C**) with VEM treatment; (**D**) saturation binding curves for Con A to AGP untreated and (**E**,**F**) treated with vemurafenib and their respective reciprocal curves (insets). Experimental conditions: PBST buffer with the addition of 1% DMSO.

The consequence of VEM influence on AGP structure should be visible in its interaction with concanavalin A. For this purpose, the QCM-D experiments were carried out using the 3rd to 13th overtones, and both changes in the frequency (Δ*f*) and the dissipation factor (Δ*D*) were recorded. After stabilization of the frequency of the modified electrode (Au/4MBA/AGP) in PBST buffer, the Con A solution in the appropriate concentration was added to the reaction chamber. This caused a rapid, significant drop in Δ*f*. This decrease was a consequence of the binding of Con A to the AGP molecules. After approximately 30 min, the frequency shift reached a stable value, which meant that the maximal amount of Con A molecules was attached to the AGP layer. The changes in the frequency shifts after addition to the QCM-D chamber of Con A at various concentrations are presented in Figure 2A–C. Moreover, it was found that the changes in the frequency shifts were found to be following a Langmuir-type adsorption isotherm (Equation (1)), as shown in Figure 2D–F.

(1) $$\Delta f=\Delta f_{\max}\frac{K_{A}\cdot C_{\mathrm{Con}\;A}}{1+K_{A}\cdot C_{\mathrm{Con}\;A}}$$

where Δ*f* represents the frequency shift, Δ*f*_max_ the maximum frequency shift at the saturation, and *K*_A_ represents the equilibrium association constant and *C*_Con A_ the concentration of concanavalin A. At saturation, the binding and dissociation rates of ligands to the available binding sites of the AGP were identical and reached a steady-state [27]. The reciprocal of this equation gives the equation of a straight line $\frac{1}{\Delta f}=\frac{1}{K_{A}\cdot C_{\mathrm{Con}\;A}\cdot \Delta f_{\max}}+\frac{1}{\Delta f_{\max}}$ from which one can derive the Δ*f*_max_ from the *y*-intercept and the value of association constant from the slope, as shown in the insets in Figure 2D–F. The equilibrium dissociation constant (*K*_D_), which is the reciprocal of *K*_A_, is obtained from Equation (2).

(2) $$K_{D}=\frac{1}{K_{A}}$$

For the regression equations describing the linear responses of $\frac{1}{\Delta f}=f\left(\frac{1}{C_{\mathrm{Con}\;A}}\right)$, the values of *K*_A_ and *K*_D_ are presented in Table 2. For all obtained dependences, the linear coefficient was not smaller than 0.991, which confirmed their usefulness in analytical analysis. The exposure of glycoprotein to VEM solution decreased its affinity towards Con A.

**Figure 3:**
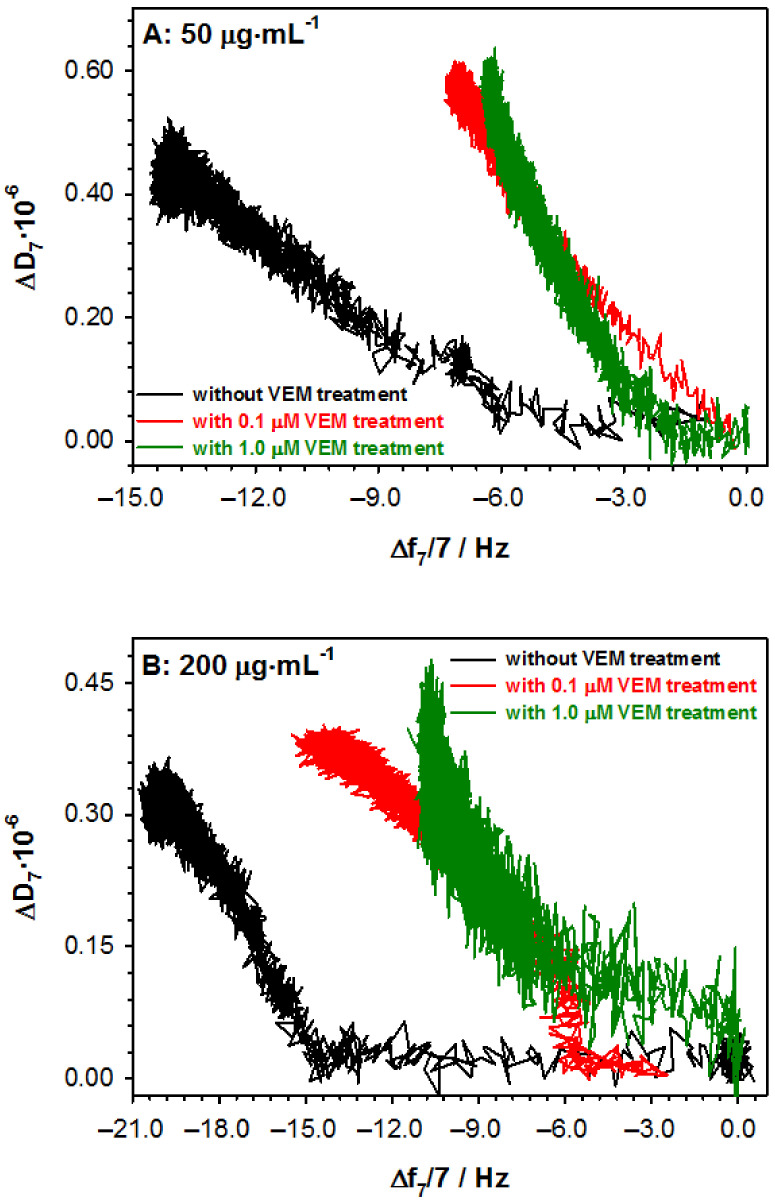
Δ*D* versus Δ*f* plots of Con A binding to AGP layer (Au/4MBA/AGP) untreated and treated with VEM for 50 (**A**) and 200 µg·mL^−1^. (**B**) Con A concentration. Experimental conditions: PBST buffer with the addition of 1% DMSO.

The D-shifts for all studied situations were positive and reached maximal values not higher than 1.0 × 10^−6^. It suggests that the formed carbohydrate-binding protein layer was densely packed. More information about the morphology of the Con A layer can be obtained from the plot of Δ*D* versus Δ*f* presented in Figure 3.

For all studied situations, two distinct regions were visible with an initial low dissipating regime followed by a higher dissipative regime. This behavior may be interpreted as a fact of the formation of the adlayer. The region in the range of the constant dissipation per frequency shift probably corresponds to the formation of the concanavalin A monolayer. Since the dimension of the subunit of concanavalin A from *Canavalia ensiformis* was 4.2 × 4.0 × 3.9 nm [28] and its molecular weight was ~44 kDa, the theoretical frequency shift corresponded to the formation of a close-packed Con A monolayer (here we used an RSA model [29] of protein adsorption) could be determined and was about −4.5 Hz. The estimated theoretical Δ*f* value (−4.5 Hz) was in good agreement with the experimental data except the 200 µg·mL^−1^ and AGP layer without VEM treatment, which confirmed the formation of Con A monolayer during the first few minutes. Moreover, the very low value of Δ*D*/Δ*f* ~5.3 × 10^−9^ Hz^−1^ proved that the formed carbohydrate-binding protein monolayer was well organized and very tight. An interesting behavior was observed for the Δ*D* = *f*(Δ*f*) dependencies for the higher dissipative regime; the slope of these curves increased in the function of the VEM concentration. This observation suggests that concanavalin A is linked to AGP layer in different ways. It should be noted that the action of VEM changed the AGP structure.

**Figure 4:**
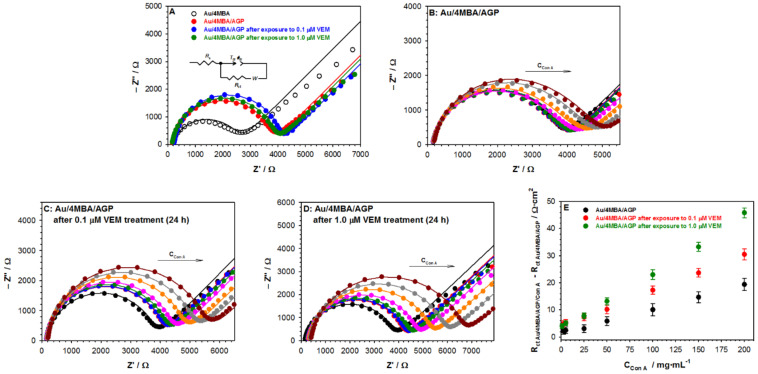
(**A**) Nyquist plots recorded during subsequent steps of electrode modification and before (white points) and (**B**–**D**) after (colored points) the interaction of Au/4MBA/GPA with ConA in various concentrations before and after VEM treatment; (**E**) Calibration plot based on EIS measurements obtained after the interaction between Con A and AGP without and with VEM treatment. Experimental conditions: PBST buffer with the addition of 1% DMSO; 5 mM Fe(CN)_6_^3-/4-^; *E*_app._ = 242 mV, Au electrode (*ϕ* = 1.6 mm). Solid lines are fitted to experimental data (points). Inset in graph A: Ershler–Randlaess equivalent circuit, where: *R*_s_–solution resistance, *R*_ct_–charge transfer resistance, *ϕ*_dl_ and *T*_dl_ are constant phase elements, *W*–Warburg impedance.

The influence of vemurafenib on the AGP structure can also be monitored by the application of electrochemical impedance spectroscopy. The typical EIS spectra recorded for subsequent steps of electrode surface modification are shown in Figure 4A. The introduction of the AGP layer into the modified electrode surface with the 4MBA layer caused a further increase in the semicircle on the Nyquist plot. The same trend was observed after the stage of AGP layer treatment with VEM and finally, after the interaction with Con A. The increase in the size of the semicircle in the Nyquist diagram was related to the increasing barrier of electron transfer between the redox probe present in the solution and the electrode surface. The EIS spectra obtained for selected Con A concentrations are shown in Figure 4B–D. In order to obtain quantitative information from the recorded EIS spectra, the theoretical curve corresponding to the equivalent circuit, presented in Figure 4A, was adjusted to the experimental curve. Table 3 shows the numerical values of the impedance parameters obtained as a result of fitting the theoretical curve to the experimental curve. Analyzing the obtained EIS parameter values, it can be seen that the layers formed during the subsequent stages of gold electrode modification were uniform and homogeneous. This was confirmed by slight changes in the value of the constant-phase element (*ϕ*_dl_), which differed by no more than 10% from the value obtained for a chemically unmodified gold electrode. During the electrode modification, the significant changes in the value of the parameters such as charge transfer resistance (*R*_ct_), Warburg parameter (*σ*), and double layer capacity (*C*_dl_) were observed compared to the chemically unmodified gold electrode surface. The values of the *R*_ct_ parameter increased with the subsequent steps of electrode surface modification. Additionally, this parameter was very sensitive to the amount of Con A interacting with AGP. As for the other parameters, such as the Warburg parameter or the capacity of the double layer, the intensity of changes in their values was much smaller and less sensitive to changes in Con A concentration. Based on changes in the Warburg parameter, it can be concluded that the transport of the redox probe underwent significant changes due to the action of vemurafenib on the AGP layer, as well as after binding the Con A to AGP molecules. Comparing the values of the double layer capacity, it was clearly visible that with the next modification, the value of this parameter decreased, and finally stabilized at the level of 8.5–10 μF cm^−2^. Such a value of *C*_dl_ indicated significant hydration of the formed layers. To visualize the changes in the degree of branching of glycans, as a result of the action of vemurafenib, the difference in the *R*_ct_ value before and after Con A–AGP interaction (Δ*R*_ct_ = *R*_ct Au/4MBA/AGP/Con A_ − *R*_ct Au/4MBA/AGP_) can be successfully used. With increasing vemurafenib concentration, the observed changes in *R*_ct_ values increased (see Figure 4E). This trend can be explained by the decrease in the degree of AGP branching, which is directly connected with the quality of the Con A layer. The small degree of branching is equivalent to a tighter and homogenous Con A layer.

The electrochemical impedance spectroscopy is known as a sensitive, real-time, non-invasive technique that can be successfully used for the detection of cytotoxicity, proliferation, and cancer cell structure changes [30,31,32,33]. Moreover, the cell-based impedance spectroscopy allows monitoring cell behavior in the presence of drugs. Such studies not only provide valuable information about drug potency and efficacy but are also crucial for drug resistance analysis [34,35]. Siedel et al. [36] proved that the multidimensional impedance platform could be successfully used in the real-time analysis of drugs in cancer cell studies. They used tissues derived from a patient to develop the 2D and 3D cell culture model for melanoma cancer. The studies showed significant differences in tissue structure responsible for BRAF inhibitor pharmacokinetics in BRAF^V600E^ tumor microfragments and cell lines. The proposed impedance approach shows great potential for quantifying drug kinetics to identify the most effective drug combinations in advanced cancer models, thereby improving personalized drug development and treatment planning, and ultimately, overall patient outcomes. Our methodology is based on the monitoring of the difference in the Rct value before and after Con A and surface cell glycoprotein interaction treated with BRAF inhibitor. The significant increase of Rct value is equivalent with successful drug action.

Methodologies used for the detection of conformational and structural changes in molecular layers or entire cells are extremely desirable in predicting cell responses to drug treatments. These capabilities are available due to such techniques as QCM-D and EIS, which allow monitoring of cell-drug interactions in real-time regiments [37,38,39,40,41]. The mass, charge transfer resistance and viscoelasticity changes of the cells caused by the drug can be used to estimate drug efficacy. In this study, we have demonstrated that the QCM-D and EIS sensing allow providing a real-time and reproducible detection of the glycoproteins structure changes induced by vemurafenib. Using different VEM concentrations, we have detected different glycoprotein patterns in response to drug exposure. At concentrations higher than 1 µM, VEM induces a QCM-D response indicating the reorganization of the glycoprotein layer. In turn, with the low VEM concentration, the QCM-D was able to sense only the fluctuations in the glycoprotein structure. Moreover, the possibility of the real-time monitoring of the glycoprotein structure changes allows understanding of the drug interaction mechanisms. The proposed methodology of the glycoprotein structure changes caused by vemurafenib, based on the interaction of glycoprotein with lectin (Con A), can be applied in the screening assays of melanoma harboring *BRAF* mutations.

### 2.2. Studies with Melanoma Cell Lines without and with BRAF Mutation

**Figure 5:**
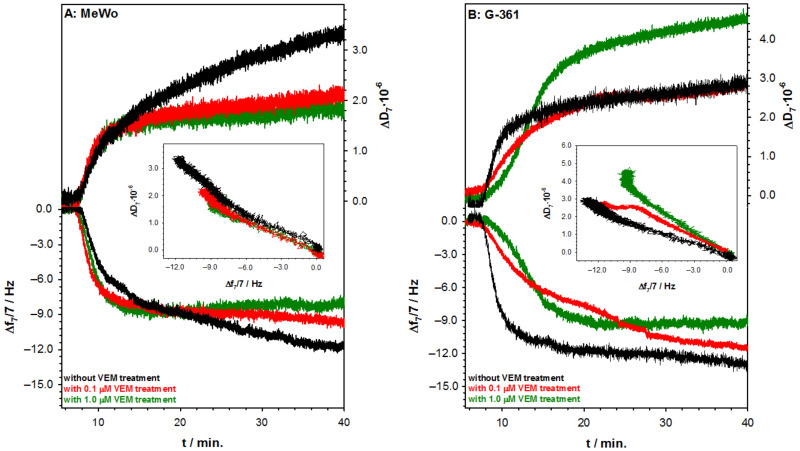
(**A**) QCM-D spectra of the shifts in frequency (Δ*f*) and dissipation factor (Δ*D*) recorded during the binding of Con A to glycans of melanoma cell lines with wild type (MeWo); (**B**) and with *BRAF* mutation (G-361) treated or untreated with VEM. Insets: Δ*D* versus Δ*f* plots of Con A binding to glycans of melanoma cell lines. Experimental conditions: PBST buffer; *C*_Con A_ = 50 µg·mL^−1^.

Many studies reveal that primary or even multiple primary melanomas differ from malignant metastases in their glycosylation profile [21,42]. It has been documented that different glycan epitopes on melanoma cells have been involved in many processes such as adhesion, motility, proliferation, angiogenesis, and immunosuppression [43]. Because glycan epitopes play significant functional roles in cancer progression and have high potential value for cancer therapy as well as diagnostics and therapies, the purpose of this study was to investigate the effect of VEM on the glycan structures in two melanoma cells lines, including those with *BRAF* mutation (G-361) and wild type (MeWo), respectively. Vemurafenib has been used as a monotherapy for the treatment of adult patients with *BRAF* V600E mutation-positive unresectable or metastatic melanoma since 2012. Interestingly, in the case of melanoma cells without these mutations, the tumor growth is not inhibited by this drug [44,45]. Therefore, in a cell line without the BRAF mutation (MeWo) the cytotoxic effects of VEM in the wide range of concentrations were not observed. In turn, the treatment of G-361 cells with VEM resulted in concentration-dependent growth inhibition of the cells (*IC*_50_ = 3.5 μM). As a tool for monitoring changes of glycan structures in melanoma cells, the lectin-concanavalin A selective binding with surface melanoma glycans was used. These changes were detected by the QCM-D measurements. Figure 5 shows typical QCM-D spectra of the frequency (Δ*f*) and dissipation factor (Δ*D*) during Con A binding to the glycans present at the melanoma cell surface. Before the interactions, the cells were treated and untreated with two selected concentrations of VEM (0.1 and 1.0 μM). The actions of VEM on the melanoma cells are well visible in the amount of associated Con A. However, the differentiation of the cells without and with *BRAF* mutation can be performed only based on the slope of the dependencies Δ*D* = *f*(Δ*f*) presented on the insets in Figure 5. The difference between slopes was seen only in the case of cells with *BRAF* mutation (G-361 cell line). The increase of the VEM concentration caused the rise of the slope of the dependence Δ*D* = *f*(Δ*f*).

**Figure 6:**
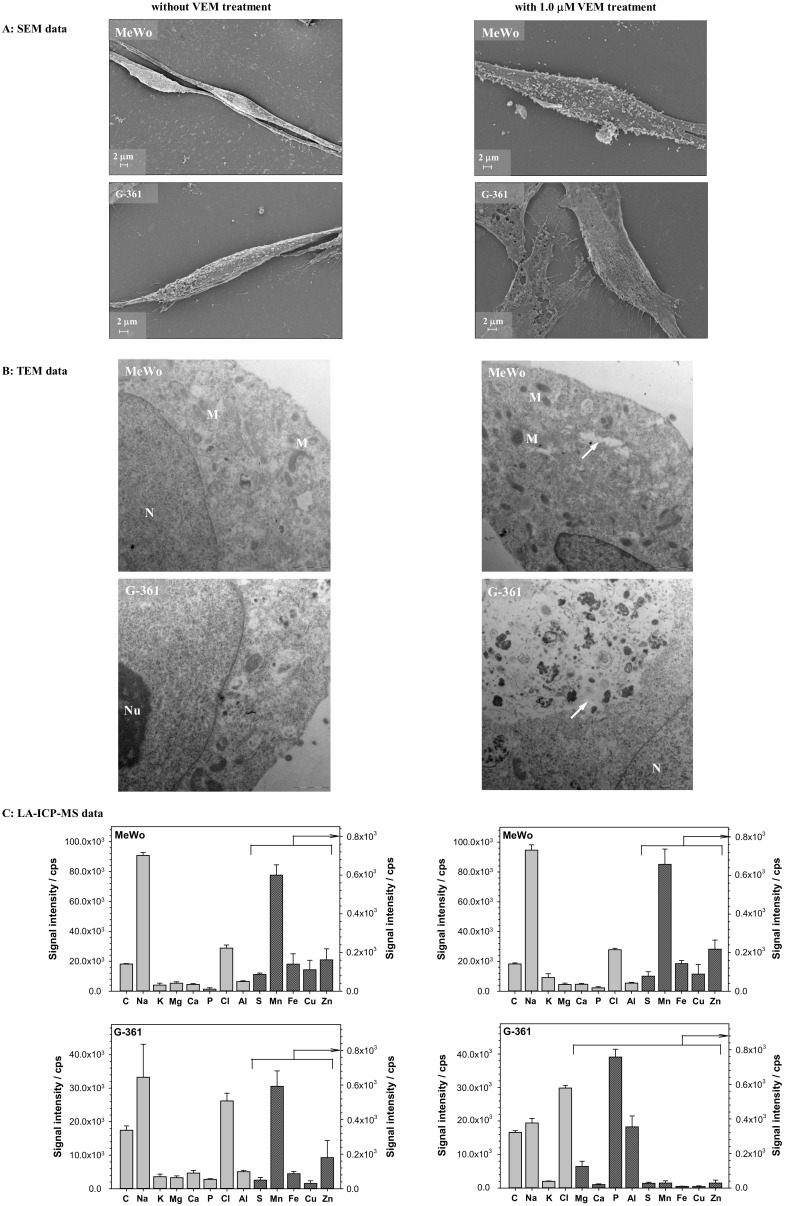
SEM (**A**), TEM (**B**) images, and LA-ICP-MS analysis (**C**) of melanoma cells without and with *BRAF* mutation before and after exposure to VEM solution.

More information about the influence of vemurafenib on the surface properties of the melanoma cells can be obtained from the scanning electron microscopy imaging. Figure 6A shows the morphology analysis of melanoma cells (MeWo and G-361) untreated and treated with 1.0 μM VEM for 24 h. Under normal growth conditions, MeWo and G-361 cells have a mixed triangular dendritic and elongated dendritic morphology. After treatment with 1.0 μM VEM for 24 h, living cells remained attached to the plate while dead cells detached. As shown by morphology analysis (right images in Figure 6A), 1.0 μM VEM induced more extensive changes in the morphology of the melanoma cells with *BRAF* mutation. The cells G-361 became less elongated, lost their spindle shape, and lost their firmness as well as elasticity. A large granularity appeared on the surface of the cells, and the cell fluid content leaked. Similar morphological changes of G-361 cells, such as loss of triangular dendritic and elongated dendritic morphology, were also observed after treatment with 0.1 μg·mL^−1^ arginine deiminase [46].

However, in the case of MeWo cells without *BRAF* mutation, the effect of vemurafenib only resulted in changes in the granularity of the cell surface, the shape was practically unchanged, and the cell fluid infiltration was also invisible. In turn, TEM analysis showed the changes caused by VEM action in the MeWo and G-361 ultrastructures. In both melanoma cell lines used in the experiment (left images in Figure 6B), organelles were typical of metabolically active cells. The cell nucleus (N) had relaxed chromatin and a big nucleolus (Nu), indicating its high transcription activity. Oval mitochondria (M) had a light grey matrix and numerous dark cristae. The remaining cell organelles had a typical ultrastructure. After treatment with 1.0 µM VEM the ultrastructural organization of MeWo cells showed visible changes (see right image in Figure 6B). Nuclear chromatin was slightly condensed, mitochondria had a denser matrix than the non-treated cells, and, in the cytoplasm, appeared electron-lucent spaces (arrow). The ultrastructure of G-361 cells after treatment with VEM also changed, but more intensively (see right image in Figure 6B). Large autophagic vacuoles were formed in the cytoplasm (arrow). Inside the vacuoles were contained numerous fragments of degrading cell’s organelles. Some cells had a disintegrated cell membrane. The increased formation of autophagic vacuoles was also observed in A-375 melanoma cells with BRAF mutation [47]. More recently, vemurafenib was shown to induce a high level of autophagy in BRAF-mutant thyroid cancer cells. The authors suggested that this process was not related to MAPK signaling pathways [48]. Interestingly, galectin-3, which belong to a superfamily of lectins, was found to decrease the level of autophagy in vemurafenib treated melanoma cells [49]. Because autophagy is recognized as a critical metabolic process involved in melanoma development and is associated with numerous molecular triggers [50], we looked further into the metallomics profiles in melanoma cells.

More specific details on vemurafenib’s effects on melanoma cells were evidenced using LA-ICP-MS measurements. In the study, the LA-ICP-MS data provided a unique pattern of the spatial metallomic heterogeneity of melanoma cells with and without *BRAF* mutation. As shown in Figure 6C, VEM action caused the decrease of Na, Ca, Mn, Fe, and Zn elements in G-361 cells, which harbors the mutation *BRAF*. This was also accompanied by slightly increased chlorine (Cl) levels. Interestingly, the increased amounts of phosphorous (P) and aluminum (Al) elements were also noted in G-361 cells treated with VEM. No such effects were observed in MeWo cells untreated and treated with VEM (Figure 6C). Melanoma cells can often acquire resistance due to membrane ATP-associated transporters that mediate direct VEM efflux [51]. Our present studies clearly evidence that VEM could affect membrane-located Na- and Ca-dependent transporters since both Na and Ca levels were diminished, elevating chloride amounts in *BRAF*-mutated G-361 cells. Because Na/K-ATP and Ca-ATPases regulate ion channels, it seems reasonable to conclude that melanoma cells with *BRAF* mutation may trigger VEM efflux in such mechanisms. A dramatic shift of phosphorous (P), which is often in the form of phosphate molecules in all cells, plays an important role in energy storage as components of ATP may also support this conclusion. Note that the most important membrane transporters in cancer cells are the ATP-dependent Na^+^, K^+^, 2Cl^−^ co-transporter NKCC1, the Na/K ATPase, cation channels, and the Na^+^/H^+^ exchanger NHE1 [52]. Therefore, it seems reasonable to suggest that changes in the size and chemical composition of the membrane glycans at the melanoma cell surface are responsible for developmental and cell-specific variability in the biophysical and functional properties of many ion channels [53]. The present study evidenced that the structure of *α*1-acid glycoprotein composing of ca. 45% of *N*-linked glycans was changed after its interaction with VEM in a dose-dependent manner (Figure 1B). Unfortunately, such mechanisms may also affect the successful clinical outcomes due to BRAF inhibitors [54,55].

Molecular studies showed that even small changes in glycan structure may regulate numerous malignancy-associated pathways, including signaling, growth, and finally, survival [55]. More recently, melanoma cells that have acquired resistance to vemurafenib elucidated elevated mitochondrial oxidative stress [56]. The authors evidenced that the oxidative profile of the resistant melanoma cells could be recognized as a potent target to overcome resistance in BRAF mutated cells [56]. Note that selective BRAF kinase inhibitor (VEM) also activates mitochondrial metabolism in BRAF-mutant human melanoma cells [57]. Because the present studies showed a significant decrease of Mn and Zn elements in G-361 *BRAF* mutated cells treated with VEM, as compared to those melanoma cells treated without BRAF kinase inhibitor, we can also associate this result with cellular manganese and zinc storage, including low molecular weight Mn^2+^ complexes not bound to enzymes and those manganese and zinc elements combined with antioxidative enzymes such as superoxide dismutase (CuZnSODs, MnSOD/FeSODs) which convert superoxide radicals (O_2_^·−^) to molecular oxygen and hydrogen peroxide (H_2_O_2_). It should be noted that zinc ions, as the vital cofactors of numerous zinc metalloproteins, impact virtually all aspects of cancer growth and metastasis [58]. Interestingly, SOD was recently found to mitigate VEM-induced superoxide radicals in *BRAF* mutant A375 cells [59]. Because VEM was shown to strongly diminish Fe, Zn, and especially Mn elements in the melanoma G-361 cells, to our knowledge, this result provides new insight into the metallomic pattern of *BRAF* mutated melanoma cells treated with and without VEM. Most melanoma patients that harbor a *BRAF* mutation develop different resistances due to VEM treatments [54,60], therefore, more detailed studies associate VEM treatment regimens, glycosylation patterns, and metallomics fingerprints are still required in *BRAF* mutated melanoma cells.

## 3. Materials and Methods

### 3.1. Materials

Concanavalin A from *Canavalia ensiformis* (Con A), *α*1-acid glycoprotein from human plasma (AGP), 4-mercaptobenzoic acid (4MBA), phosphate buffered saline (PBS), dimethyl sulfoxide (DMSO), polyethylene glycol sorbitan monolaurate (Tween-20), *N*-hydroxysuccinimide (NHS), *N*-(3-dimethylaminopropyl)-*N*′-ethylcarbodiimide hydrochloride (EDC), glutaraldehyde, osmium tetroxide solution (OsO_4_ in bidistillated water), cacodylic acid (≥98%), and acetone were purchased from Sigma-Aldrich, Poznań, Poland; ethanol (≥99.8%) was purchased from Avantor, Gliwice, Poland, and vemurafenib (VEM) was purchased from Selleckchem, Poznań, Poland. All were used without additional purification. All solutions were prepared with ultrapure water (Hydrolab, conductivity 0.056 μS·cm^−1^). The measurements were performed in PBST buffer (PBS with Tween-20 (0.025% *v*/*v*)), pH 7.4 with 1% addition of DMSO.

### 3.2. Cell Culture

The human melanoma cell lines G-361 (with *BRAF* mutation) and MeWo (without *BRAF* mutation) were obtained from the American Type Culture Collection (ATCC, Manassas, VA, USA).

The cultures were cultivated in a 5% CO_2_ atmosphere at 37 °C in a CO_2_ incubator (Memmert, Schwabach, Germany). The cells were grown as adherent monolayers in F-12K Medium (Kaighn’s Modification of Ham’s F-12 Medium, Gibco, Paisley, UK), supplemented with 10% FBS (Fetal Bovine Serum, Gibco, Paisley, UK) and antibiotics: streptomycin, 10.000 μg·mL^−1^ and penicillin, 10.000 U·mL^−1^ (Gibco, Paisley, UK). The medium was changed every 3rd day. For subculture, the cells were washed twice with phosphate-buffered saline (PBS) and incubated with trypsin-EDTA solution (0.05% trypsin, 1 mM EDTA) for 2 min at 37 °C to detach the cells. Next, to inhibit the action of trypsin, the complete media were added. The cells were washed twice by centrifugation and resuspended in the complete fresh media for reseeding and growing in new culture flasks or plates. Cells were counted using a hemocytometer.

### 3.3. Quartz Crystal Microbalance with Dissipation (QCM-D)

The experiments were performed with a QCM E4 instrument (Q-sense AB, Sweden) equipped with 4.95 MHz quartz crystals coated with gold (type QSX 301 Gold used in the AGP studies) and polystyrene (QSX 305 PS used in the cells studies). Before the experiments, the Au crystals were cleaned in TL1 mixture (ultrapure water, 25% ammonia, and 30% hydrogen peroxide in the volume ratio 5:1:1) and heated to temperature 75 °C, by immersing them for 5 min. Next, the surface of the electrodes was rinsed with ultrapure water and dried with Ar stream. Before anchoring the AGP, the gold sensors were firstly modified by SAM layer (Au/4MBA). To form a 4MBA thiol layer, the gold sensor was immersed in 1 mM 4MBA ethanolic solution overnight at room temperature. After that time, the sensor surface was carefully rinsed with pure ethanol and water to remove physically adsorbed thiol molecules. Next, the carboxylic groups of thiol chains were activated for 1 h with a standard aqueous mixture of EDC/NHS (40 mM/10 mM). Finally, the sensor was incubated in 0.2 mg·mL−1 AGP solution for 1 h to form the glycoprotein layer at the Au/4MBA. The modified sensor (Au/4MBA/AGP) was then ready to use.

In turn, before the experiments with melanoma cells, polystyrene coated quartz crystals were immersed in 1% deconex (Borer ChemieAG,) for 30 min at 65 °C. Thereafter, sensors were kept in Hydrolab water for at least 2 h and dried with argon. Before seeding the cells, the PS-sensors were sterilized in 70% ethanol for 20 min and next washed with a PBS buffer for 15 min. The cells were seeded at a density of 5 × 104 cells per cm2 supplemented with the nutrient solution on the PS sensors and incubated for 24 h. Next, the medium was replaced with the appropriate VEM concentration in the full growth medium and incubated for an additional 24 h. Before the measurements, sensors with cells were washed with PBS buffer, fixed with 3.7% paraformaldehyde in PBS, and inserted into the QCM-D chamber. The QCM-D measurements (n = 3) were carried out in the flow system with a flow rate of 25 μL·min−1 and a constant temperature of 37 °C.

### 3.4. Electrochemical Impedance Spectroscopy (EIS)

The measurements were performed in the three-electrode system consisting of (i) working electrode (gold disc electrode; 1.6 mm in diameter, BASi, Tetbury, England), (ii) reference electrode (Ag/AgCl/3 M KCl), and (iii) auxiliary electrode (Pt plate with surface area at least 1 cm^2^) with using an Autolab, model PGSTAT 12 potentiostat. Impedance spectra were recorded in the frequency range from 0.01 Hz to 10 kHz with the ac amplitude equal to 5 mV (peak-to-peak). The fitting of the experimental data to the appropriate equivalent circuit was performed using the complex nonlinear least-squares (CNLS) method. Each time, before the measurements, the surface of the working electrode was polished on a wet pad with the addition a 1 and 0.3 μm Al_2_O_3_ powder. Each electrode after polishing was rinsed with a direct stream of ultrapure water (Hydrolab, conductivity of ~0.056 μS·cm^−1^) to remove alumina completely from the electrode surface. Then it was dried with argon and modified with the 4MBA and AGP layers (the procedure of modification the same as for Au sensors). In all experiments, the electrochemical cell was kept in a Faraday cage to minimize the electrical noise.

### 3.5. Transmission Electron Microscopy (TEM) and Scanning Electron Microscopy (SEM) Imaging

For analysis in a TEM, the cells were dissociated using trypsin/EDTA saline and fixed in 2.5% glutaraldehyde in 0.1 M cacodylate buffer for 24 h. The postfixation took place in 1% OsO_4_ in bidistillated water for 6 h. Afterward, the samples were dehydrated by ethanol solutions of increasing concentration (30%–10 min, 50%–10 min, 70%–24 h, 90%–10 min, 96%–10 min, anhydrous EtOH–10 min, finally acetone–10 min). The cells to be analyzed in SEM were dried at a critical point, and when dry, the monolayer was excised from the tissue culture dish. The disc was then mounted to an aluminum stub, and the samples were sputter-coated with a gold alloy. Following sputter coating, the samples were viewed with a scanning electron microscope (FE-SEM, Merlin, Zeiss, Oberkochen, Germany). After fixation and dehydration, the cells to be analyzed in TEM, were saturated with epone (first mixed with successive acetone solutions of increasing concentration (1:3–30 min, 1:1–2 h, 3:1–5 h) and then pure (for 12 h). After saturation, the epone was polymerized in blocks at 60 °C for 24 h in an incubator (Agar Scientific, Stansted, England). The polymerized samples were cut into ultrathin sections (70 nm thick) with an RMC MTX ultramicrotome (Boeckeler Instruments, Tucson, Arizona, USA), placed onto copper nets, contrasted with uranyl acetate and Reynolds reagent [61] and analyzed in a LIBRA 120 transmission electron microscope produced by Carl Zeiss (Oberkochen, Germany). Photographs were made with a Slow-Scan CCD camera (ProScan, Germany), using the EsiVision Pro 3.2 software.

For SEM examination, low kV electron beam energy was used (3 kV, 30 pA current). Before the examination, each sample was covered with a 1–2 nm thin film of Au-Pd alloy to avoid electrical charging of the sample surface. The layers of the alloy were sputtered using a Polaron SC7620 Mini Sputter Coater. Elemental analysis (energy dispersive X-ray spectrometry (EDS)) was carried out using multichannel device EDS XFlash Detector 5010 125 eV, Quantax (Bruker, Mannheim, Germany) using a 10 kV electron beam energy. Measurements were made on non-sputtered samples.

### 3.6. ICP-MS Measurements with Laser Ablation (LA-ICP-MS)

An Inductively Coupled Plasma Mass Spectrometer (Perkin Elmer NexION 300, Waltham, Massachusetts, USA) equipped with a laser ablation system (LSX-213, CETAC, Omaha, Nebraska, USA) was used. The UV laser (Nd-YAG, solid-state, *λ* = 213 nm) ablation was operated at a constant 10 Hz repetition rate, the energy of 5.0 mJ·pulse^−1^, and a spot size of 150 µm. The signal intensities were registered for the 13 selected isotopes: ^12^C, ^23^Na, ^39^K, ^43^Ca, ^26^Mg, ^31^P, ^32^S, ^35^Cl, ^27^Al, ^57^Fe, ^55^Mn, ^65^Cu, and ^66^Zn, using the peak hopping mode and 10 ms dwell time. All measurement cycles consisted of the signal intensities registration during the multi-line ablation (*n* = 3) over the area selected on the surface of the sample. The signals of blank values registered for 15 s before the start of ablation for each selected isotope were subtracted from the signals recorded during the ablation of the samples. All experiments were performed using Ar as the carrier gas.

### 3.7. Statistical Analysis

For each QCM-D, EIS, LA-ICP-MS, SEM, and TEM measurements, three independent experimental repetitions (*n* = 3) were performed. Data are shown as mean ± SD, and they are representative of three replicates. The student’s *t*-test was performed to determine significant differences between the tested parameters. The difference was considered significant if *p* < 0.05.

## 4. Conclusions

Alpha-1-acid glycoprotein is a prominent example of a molecule in which alterations in the structure of the surface oligosaccharides caused by anticancer drugs such as vemurafenib, a selective inhibitor of BRAF kinase, can be a great predictor in the diagnosis and determination of the effectiveness of treatments in patients with advanced melanoma harboring a *BRAF* mutation. The present studies show that the changes in the glycans structure due to VEM treatments can be easily detected on the basis of their interaction with lectin (Con A) using quartz crystal microbalance with dissipation and electrochemical impedance spectroscopy. Furthermore, these data demonstrate that such changes in the AGP structure caused by this BRAF kinase inhibitor can affect a global change in the cell surface glycosylation pattern that significantly alters melanoma cell growth, survival, and plausibly resistance performances.

The most prominent achievement of this work is the proof for the first time that vemurafenib strongly modulates the metallomics profile in *BRAF* mutated melanoma cells. Our present studies evidence that VEM affecting the glycan structures on *BRAF* mutated cells could affect the membrane-associated transporters targeting numerous other molecular endpoints. Why this could also directly trigger the resistance phenomenon in malignant melanoma is still open debate and worthy of further exploration.

## Figures and Tables

**Table 1:** EIS parameters (*n* = 3) (capacitive parameter-*T*_dl_ and exponential factor-*ϕ*_dl_ of the constant phase element (CPE); charge transfer resistance-*R*_ct_), Warburg parameter-*σ*, and double layer capacity-*C*_dl_) for each step of electrode modification and VEM interactions with AGP.

Electrode Modyfication	*T*_dl_ [μF·s^(1−*ϕ*)^·cm^−2^]	*ϕ*_dl_	*R*_ct_ [Ω·cm^2^]	*σ* [Ω·rad^1/2^·s^−1/2^·cm^2^]	*C*_dl_ [μF·cm^−2^]
Au	82.1 ± 0.4	0.91 ± 0.02	6.5 ± 0.2	25.2 ± 0.3	23.0 ± 0.9
Au/4MBA	98.2 ± 2.2	0.87 ± 0.04	48.6 ± 2.2	39.0 ± 1.1	13.5 ± 0.4
Au/4MBA/AGP	33.4 ± 1.2	0.91 ± 0.03	72.8 ± 4.3	39.1 ± 2.5	12.8 ± 0.5
Au/4MBA/AGPafter exposure to 0.1 μM VEM	20.3 ± 3.5	0.92 ± 0.04	75.6 ± 5.2	38.5 ± 2.3	11.9 ± 1.0
Au/4MBA/AGPafter exposure to 1.0 μM VEM	20.4 ± 2.5	0.94 ± 0.03	77.8 ± 3.2	36.7 ± 2.4	9.0 ± 1.1

**Table 2:** Calibration equations and values of *K*_A_ and *K*_D_ for AGP layer untreated and treated with VEM.

Au/4MBA/AGP	Regression Equation	*R* ^2^	*K*_A_/[M^−1^]	*K*_D_/[M^−1^]
without VEM treatment	$\frac{1}{\Delta f}=\left(1.18\pm 0.04\right)\times 10^{-8}\frac{1}{C_{\mathrm{Con}\;A}}+\left(4.51\pm 0.10\right)\times 10^{-2}$	0.998	3.82 × 10^6^	2.61 × 10^−7^
with 0.1 μM VEM treatment	$\frac{1}{\Delta f}=\left(3.31\pm 0.23\right)\times 10^{-8}\frac{1}{C_{\mathrm{Con}\;A}}+\left(8.00\pm 0.60\right)\times 10^{-2}$	0.991	2.42 × 10^6^	4.13 × 10^−7^
with 1.0 μM VEM treatment	$\frac{1}{\Delta f}=\left(3.82\pm 0.15\right)\times 10^{-8}\frac{1}{C_{\mathrm{Con}\;A}}+\left(5.14\pm 0.38\right)\times 10^{-2}$	0.997	1.34 × 10^6^	7.46 × 10^−7^

**Table 3:** EIS parameters (*n* = 3) (capacitive parameter-*T*_dl_ and exponential factor-*ϕ*_dl_ of the constant phase element (CPE); charge transfer resistance-*R*_ct_), Warburg parameter-*σ*, and double layer capacity-*C*_dl_) for each step of electrode modification and Con A interactions.

Concentartion of Con A [μg·mL^−1^]	*T*_dl_ [μF·s^(1−*ϕ*)^·cm^−2^]	*ϕ*_dl_	*R*_ct_ [Ω·cm^2^]	*σ* [Ω·rad^1/2^·s^−1/2^·cm^2^]	*C*_dl_ [μF·cm^−2^]
**Au/4MBA/AGP**					
2.5	32.2 ± 3.6	0.88 ± 0.01	74.6 ± 2.7	28.8 ± 2.7	9.2 ± 0.8
5.0	32.0 ± 2.3	0.88 ± 0.02	75.2 ± 1.2	29.0 ± 2.2	9.2 ± 0.6
25	31.8 ± 2.7	0.88 ± 0.01	75.6 ± 1.4	29.2 ± 2.1	9.1 ± 0.8
50	30.5 ± 1.3	0.88 ± 0.03	78.6 ± 2.6	30.3 ± 2.5	8.7 ± 0.4
100	28.8 ± 2.6	0.89 ± 0.02	83.4 ± 2.7	32.2 ± 2.6	9.2 ± 0.7
150	27.2 ± 1.6	0.89 ± 0.02	88.5 ± 2.7	34.1 ± 2.6	8.8 ± 0.7
200	25.9 ± 2.3	0.89 ± 0.02	92.9 ± 2.7	35.8 ± 2.2	8.4 ± 0.8
**Au/4MBA/AGP after exposure to 0.1 μM VEM**					
2.5	22.6 ± 1.6	0.94 ± 0.01	78.4 ± 2.7	46.9 ± 2.7	12.5 ± 0.8
5.0	22.5 ± 2.3	0.94 ± 0.02	78.8 ± 1.2	47.1 ± 3.2	12.5 ± 0.6
25	21.8 ± 1.7	0.94 ± 0.01	81.0 ± 1.4	48.4 ± 2.1	12.1 ± 0.8
50	21.0 ± 1.3	0.95 ± 0.03	84.2 ± 3.6	50.4 ± 2.5	12.8 ± 0.4
100	19.3 ± 1.6	0.95 ± 0.02	91.9 ± 2.7	54.9 ± 2.6	11.8 ± 0.7
150	18.0 ± 1.6	0.95 ± 0.02	98.7 ± 2.7	58.9 ± 2.6	11.0 ± 0.7
200	16.8 ± 1.3	0.95 ± 0.02	105.7 ± 5.7	63.2 ± 2.2	10.3 ± 0.8
**Au/4MBA/AGP after exposure to 1.0 μM VEM**					
2.5	25.0 ± 1.6	0.92 ± 0.01	79.6 ± 2.7	37.2 ± 2.7	11.5 ± 0.8
5.0	24.7 ± 1.3	0.92 ± 0.02	80.6 ± 3.2	37.6 ± 3.2	11.5 ± 0.6
25	23.8 ± 1.7	0.92 ± 0.01	83.4 ± 2.4	39.0 ± 3.1	11.0 ± 0.8
50	22.5 ± 1.3	0.93 ± 0.03	89.3 ± 2.6	41.8 ± 2.5	11.4 ± 0.4
100	20.0 ± 1.6	0.93 ± 0.02	100.1 ± 2.7	46.7 ± 2.6	10.3 ± 0.7
150	18.0 ± 1.6	0.94 ± 0.02	110.9 ± 2.7	51.9 ± 2.6	10.2 ± 0.7
200	16.0 ± 1.3	0.95 ± 0.02	124.6 ± 4.7	58.3 ± 1.2	9.5 ± 0.8

## Data Availability

The data presented in this study are available on request from the corresponding author. The data are not publicly available due to privacy.

## Abbreviations

AGP	*α*1-acid glycoprotein
Con A	*Canavalia ensiformis*
DMSO	dimethyl sulfoxide
EIS	electrochemical impedance spectroscopy
LA-ICP-MS	inductively coupled plasma mass spectrometer with laser ablation
PBST	phosphate-buffered saline, 0.1% Tween 20 detergent
SEM	scanning electron microscopy
TEM	transmission electron microscopy
QCM-D	quartz crystal microbalance with dissipation
VEM	vemurafenib
